# Delayed diagnosis of Lemierre’s syndrome in a patient with severe coronavirus disease 2019: importance of comprehensive oral and neck examination – a case report

**DOI:** 10.1186/s12879-023-08755-2

**Published:** 2023-11-07

**Authors:** Tomotaka Miura, Hirotsugu Fukuda, Hiroshi Kawada, Tetsuro Kaga, Masayuki Matsuo, Tsutomu Sakai, Shozo Yoshida, Hideshi Okada, Shinji Ogura, Nobuyuki Tetsuka

**Affiliations:** 1 Advanced Critical Care Center, Gifu University Hospital, Gifu, Japan; 2 Department of Infection Control, Gifu University Graduate School of Medicine, 1-1 Yanagido, Gifu-Shi, Gifu, 501-1194 Japan; 3 Department of Radiology, Gifu University, Gifu, Japan; 4 Gastroenterology, Hashima City Hospital, Gifu, Japan

**Keywords:** Case report, Coronavirus disease 2019, Interventional radiology, Lemierre’s syndrome, Pulmonary artery pseudoaneurysm

## Abstract

**Background:**

Given the widespread prevalence of the coronavirus disease 2019 (COVID-19), oral and neck examinations tend to be avoided in patients with suspected or confirmed COVID-19. This might delay the diagnosis of conditions such as Lemierre’s syndrome, which involves symptoms resembling COVID-19-related throat manifestations.

**Case presentation:**

A 24-year-old man without any underlying conditions was diagnosed with COVID-19 7 days before presentation. He was admitted to another hospital 1 day before presentation with severe COVID-19 and suspected bacterial pneumonia; accordingly, he was started on treatment with remdesivir and meropenem. Owing to bacteremic complications, the patient was transferred to our hospital for intensive care. On the sixth day, the patient experienced hemoptysis; further, a computed tomography (CT) scan revealed new pulmonary artery pseudoaneurysms. Successful embolization was performed to achieve hemostasis. In blood cultures conducted at the previous hospital, *Fusobacterium nucleatum* was isolated, suggesting a cervical origin of the infection. A neck CT scan confirmed a peritonsillar abscess and left internal jugular vein thrombus; accordingly, he was diagnosed with Lemierre’s syndrome. The treatment was switched to ampicillin/sulbactam, based on the drug susceptibility results. After 6 weeks of treatment, the patient completely recovered without complications.

**Conclusion:**

This case highlights the significance of thorough oral and neck examinations in patients with suspected or diagnosed COVID-19 for the detection of throat and neck symptoms caused by other conditions.

## Background

Lemierre’s syndrome is characterized by septic thrombophlebitis of the internal jugular vein, typically due to pharyngeal infection. It is characterized by inflammation of the vessel wall, resulting in the formation of an infected thrombus within the venous lumen and progression to surrounding soft tissue inflammation. Lemierre’s syndrome is frequently accompanied by persistent bacteremia and septic emboli, with the lungs being the most common sites of distant metastasis [1]. Its clinical manifestations include fever, sore throat, dysphagia, and respiratory symptoms; however, there is a rare occurrence of hemoptysis. Given the time lag between metastatic progression and lung involvement, pharyngeal symptoms may subside by the time respiratory symptoms, including respiratory distress, become apparent. Diagnosis is often established by the detection of *Fusobacterium* species*,* which is the most common pathogen found in blood cultures [2, 3].

The coronavirus disease 2019 (COVID-19) pandemic has significantly affected routine medical practices [4]. To mitigate the risk of exposure to severe acute respiratory syndrome coronavirus 2 (SARS-CoV-2) among healthcare providers, the use of personal protective equipment has been emphasized when examining patients with respiratory symptoms. Furthermore, examinations of the oral cavity and neck region are avoided in patients with confirmed COVID-19 to minimize exposure to SARS-CoV-2. However, this has delayed the diagnosis of conditions such as Lemierre’s syndrome, which is characterized by symptoms resembling COVID-19-related throat manifestations. This article describes a case of Lemierre’s syndrome diagnosed in a patient with COVID-19.

## Case presentation

A 24-year-old man presented with a fever 7 days prior to presentation and was diagnosed with COVID-19 according to the result of an antigen test. One day before presentation, he visited a municipal hospital with a complaint of dyspnea, with a subsequent SARS-CoV-2 antigen test yielding a positive result. Chest computed tomography (CT) revealed diffuse areas of pulmonary nodular opacities, with some of them exhibiting cavities. The patient met the criteria for severe illness based on the Japanese Guidelines for Clinical Management of Patients with COVID-19, consistent with the classification of severe disease according to the World Health Organization guidelines [5]. Subsequently, the patient was admitted and started on remdesivir. After collecting samples for two sets of blood cultures, meropenem was administered as a precautionary measure against potential complications of bacterial pneumonia. Sputum samples were not obtained owing to concerns related to COVID-19. The next day, the blood cultures yielded positive results. At this stage, only the anaerobic culture was positive; further, the specific bacterial species remained unidentified. Subsequently, the patient was transferred to our university hospital for further investigation and specialized management.

The patient had no relevant medical history. He worked as a tavern clerk and had a history of heavy alcohol consumption. He had never smoked or taken any medication. The patient had received a series of three COVID-19 vaccinations.

On admission to our hospital, the patient presented with fever, fatigue, dyspnea, and cough. He denied experiencing sore throat, neck pain, difficulty in swallowing, hoarseness, chest pain, or abdominal pain. The patient was 161 cm tall and weighed 61 kg. He was conscious and alert with a body temperature of 37.8 °C, heart rate of 130 beats per minute, blood pressure of 152/77 mmHg, respiratory rate of 30 breaths per minute, and oxygen saturation of 95%, with mask oxygenation at 8 L/min. There were no remarkable physical examination findings; however, he did not undergo pharyngeal examination given his critical condition due to COVID-19. Table 1 shows the laboratory examination results. The Sequential Organ Failure Assessment score on the admission day was 8, with a score of 3 each for respiration and coagulation and 1 each for liver and renal function. Accordingly, the patient was diagnosed with severe COVID-19 accompanied by anaerobic bacteria-induced bacteremia. He received remdesivir, dexamethasone, and heparin for COVID-19 as per the Japanese Guidelines for Clinical Management of Patients with COVID-19, along with continued meropenem administration for bacteremia.

**Table 1:** Laboratory findings at the time of transfer to our hospital

**Biochemical tests**			
TP	5.7 g/dL	Na	122 mmol/L
Alb	2.1 g/dL	K	3.2 mmol/L
CK	573 U/L	Cl	85 mmol/L
AST	122 U/L	Ca	6.7 mg/dL
ALT	85 U/L	T-Bil	1.8 mg/dL
LD	537 U/L	D-Bil	1.2 mg/dL
ALP	146 U/L	CRP	32.5 mg/dL
γGTP	107 U/L	PCT	> 100 ng/mL
ChE	116 U/L	β-D glucan	< 6.0 pg/mL
Amy	69 U/L	HbA1c	5.9%
Cre	1.38 mg/dL		
BUN	38.2 mg/dL		
**Blood** **cell** **count**		**Coagulation** **test**	
WBC	25,820 /uL	APTT	30.9 sec
Hb	13.1 g/dL	PT	78%
Plt	41,000 /uL	FDP	9.7 ug/mL
**Blood gas analysis (8 L/min oxygen mask)**			
pH	7.435	HCO3-	22.4 mmol/L
PaCO2	33.9 mmHg	Lac	13 mg/dL
PaO2	68.2 mmHg	BE	-0.8

*TP* Total protein, *Alb* Albumin, *AST* Aspartate transaminase, *ALT* Alanine transaminase, Lactate dehydrogenase, *ALP* Alkaline phosphatase, *γGTP* γ-glutamyltransferase, *ChE* Cholinesterase, *Amy* amylase, *Cre* Creatinine, *BUN* Blood urea nitrogen, *T-Bil* Total bilirubin, *D-Bil* Direct bilirubin, *CRP* C-reactive protein, *PCT* Procalcitonin, *HbA1c* Hemoglobin A1c, *WBC* White blood cell, *Hb* Hemoglobin, *Plt* Platelet, *APTT* Activated partial thromboplastin time, *PT* Prothrombin time, *FDP* Fibrinogen/fibrin degradation products, *pH* Potential hydrogen, *PaCO2* Partial pressure of carbon dioxide, *PaO2* Partial pressure of oxygen, *HCO3* Bicarbonate ion, *Lac* Lactate, *BE* Base excess

**Fig. 1:**
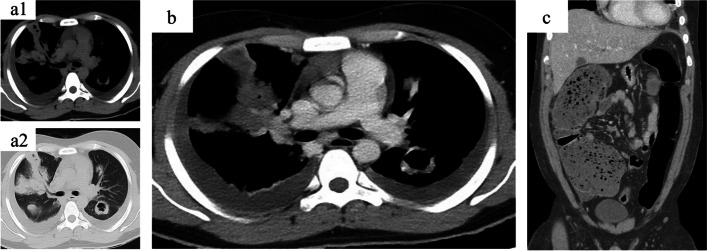
Contrast-enhanced computed tomography on day 6 of hospitalization. Multiple infiltrative shadows with associated cavities were observed (1-a1 & 1-a2). No pulmonary artery aneurysm was detected (1-b). The abdominal scan revealed a dilated ascending colon (1-c)

Although the exact cause of the bacteremia remained unclear, the patient’s overall condition gradually improved. On the fourth hospitalization day, his oxygen saturation reached 98% while receiving oxygen support via a nasal cannula at a rate of 2 L/min. The Sequential Organ Failure Assessment score was 2, with a score of 2 for respiration. Figure 1 shows the contrast-enhanced computed tomography (CECT) scans of the chest, abdomen, and pelvis. Specifically, the chest CECT scans exhibited a similar pattern to that observed 5 days prior, while the abdomen CECT scans revealed a dilated ascending colon. Abscesses and pulmonary artery pseudoaneurysms were not observed. A provisional diagnosis of bacterial translocation from the ascending colon to septic pulmonary emboli was established.

**Fig. 2:**
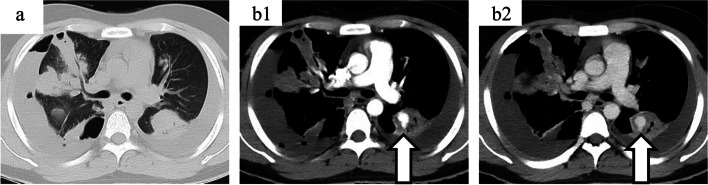
Chest contrast-enhanced computed tomography (CT) on day 8 of hospitalization. Figure 2-a shows a plain CT scan, while Figure 2-b1 and Figure 2-b2 depict the early and late contrast-enhanced phases, respectively. A newly developed right pneumothorax was observed (2-a), along with a pulmonary artery pseudoaneurysm in the left A6 (2-b1 & 2-b2)

**Fig. 3:**
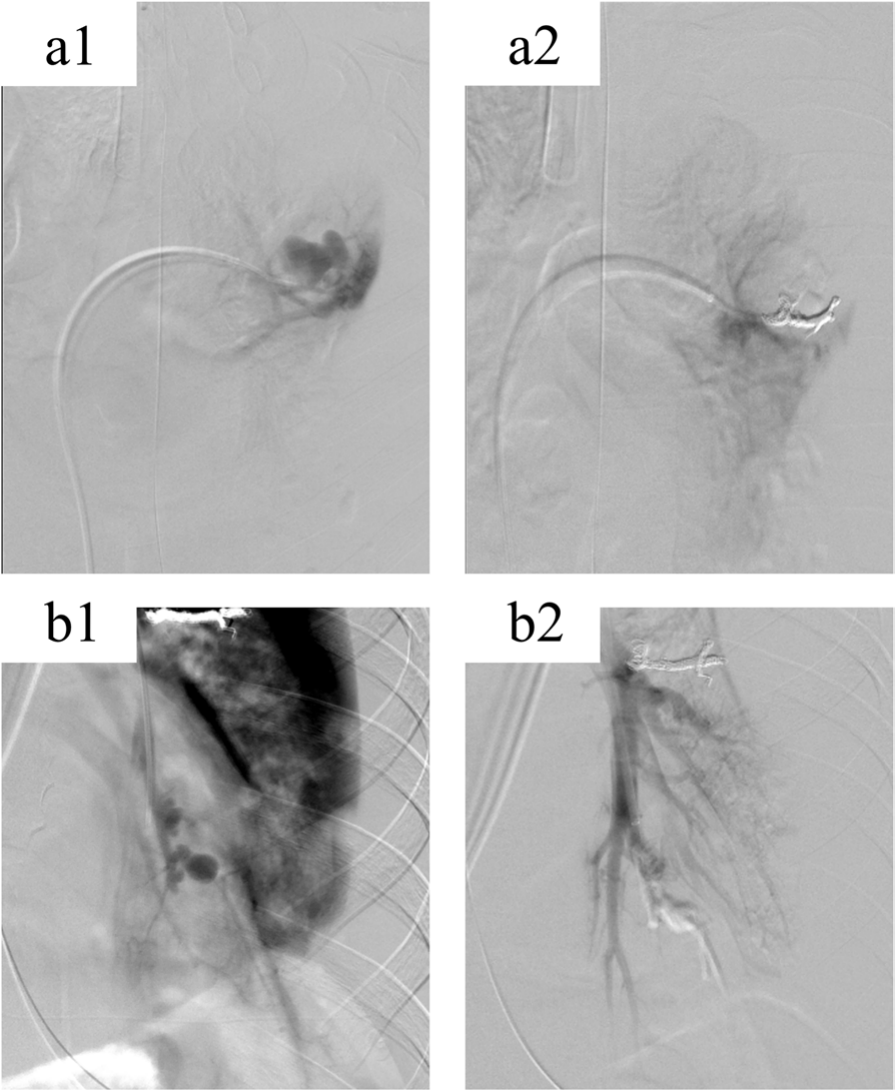
Pulmonary angiography and embolization of pulmonary artery pseudoaneurysms. Pseudoaneurysms were identified in the left A6 (3-a1) and A9 (3-b1) arteries. We successfully performed embolization of these pseudoaneurysms using coils and n-butyl-2-cyanoacrylate (3-a2 & 3-b2)

On the sixth day of hospitalization, the patient experienced a sudden episode of hemoptysis. A chest CECT revealed increased bilateral pleural effusion. Moreover, we observed a pulmonary artery pseudoaneurysm in the left superior segmental artery (A6) and newly developed right pneumothorax (Fig. 2). Given the risk of exacerbating infection after embolization or surgery, we decided to discontinue heparin and closely monitor the patient’s condition. However, hemoptysis recurred and his respiratory status deteriorated on the seventh day of hospitalization. Therefore, ventilator management was initiated, and bilateral chest drainage tubes were inserted. Endovascular therapy was started given the patient’s overall condition. Pulmonary arteriography revealed two pulmonary pseudoaneurysms in the left interlobar artery (A6 [also identified on contrast CT] and A9 [identified on pulmonary arteriography but not on contrast CT]) (Fig. 3). Initially, we planned to perform aneurysm embolization only using n-butyl-2-cyanoacrylate (NBCA). However, due to difficulties in catheter stabilization, embolization was performed using a combination of a metallic coil and a 25% NBCA mixture, with flow control using a balloon-guided catheter. Successful embolization resulted in hemostasis.

**Fig. 4:**
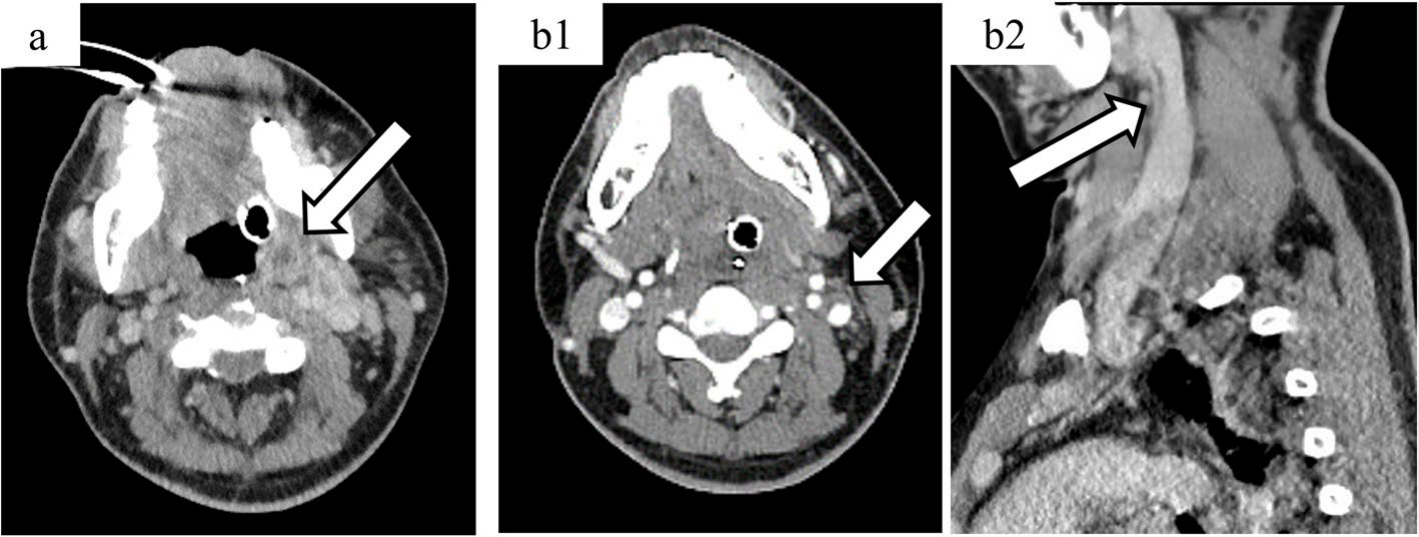
Neck contrast-enhanced computed tomography on day 10 of hospitalization. A peritonsillar abscess (4-a) and thrombus in the left internal jugular vein (4-b1 & 4-b2) were observed

On the eighth day of hospitalization, we received a report from the previous hospital that indicated the presence of *F. nucleatum* in blood cultures obtained 1 day before admission to our hospital. Since *F. nucleatum* is a commensal anaerobic bacterium commonly found in the oropharynx and pharynx, we postulated that the patient developed bacteremia due to *F. nucleatum* originating from the head and neck region. Subsequent CECT scans of the head and neck region revealed the presence of a peritonsillar abscess and thrombus in the left internal jugular vein (Fig. 4). Accordingly, we established a definitive diagnosis of Lemierre’s syndrome. Subsequent blood and pleural effusion cultures obtained at our hospital revealed no microorganism growth.

Accordingly, the patient was switched from meropenem to ampicillin/sulbactam. The patient experienced no further hemoptysis, and his respiratory status steadily improved. Extubation was successfully performed on the 17^th^ day of hospitalization. The left and right chest tubes were removed on the 17^th^ and 24^th^ days, respectively. On the 25^th^ day, edoxaban tosylate hydrate was started to treat the thrombus in the internal jugular vein. Head magnetic resonance imaging and angiography performed on the 21^st^ day of hospitalization revealed normal findings. Subsequent CECT from the neck to the pelvis on the 27^th^ day revealed a reduction in the size of the peritonsillar abscess, internal jugular vein thrombus, and pulmonary opacities. Notably, there was no evidence of new pseudoaneurysm or abscess formation.

On the 30^th^ day of hospitalization, the patient was transferred to a municipal hospital and was continued on ampicillin/sulbactam and edoxaban tosylate hydrate. After 6 weeks of antimicrobial therapy, he was discharged without any complications.

## Discussion and conclusions

Lemierre’s syndrome is a rare condition that primarily affects young, otherwise healthy adults [6]. It is characterized by metastatic infections caused by thrombophlebitis of the internal jugular vein [3]. The most common pathogenic agents are *Fusobacterium* species, including *F. necrophorum* and *F. nucleatum*. These are anaerobic gram-negative rods that are normal inhabitants of the oral cavity [3]. Generally, the clinical course of Lemierre’s syndrome can be classified into three stages. The first stage is characterized by pharyngitis. The second stage involves local invasion of the lateral pharyngeal space and internal jugular veins. The third and final stage involves metastatic complications [2]. These complications primarily manifest as septic embolisms, with the lungs being the most commonly affected site [1]. Lemierre’s syndrome is often detected during advanced stages of metastatic complications [2, 3]. Similar to our case, the detection of *Fusobacterium* species in blood cultures is a key indicator of Lemierre’s syndrome [2].

The initial CECT scan did not include the neck region, which could have allowed earlier detection of the internal jugular vein thrombus. In the backdrop of the COVID-19 outbreak, pharyngeal examinations are often avoided; however, it is important to carefully consider the possibility of pharyngolaryngeal infection even in patients with confirmed COVID-19. We emphasize the importance of conducting physical examinations and blood cultures for accurate diagnosis.

The findings of this case report are unique for several reasons. First, this case report describes the occurrence of Lemierre’s syndrome complicated by COVID-19. COVID-19 and Lemierre’s syndrome have similar symptoms, indicating the importance of not overlooking Lemierre’s syndrome during the COVID-19 pandemic [7]. There have been previous reports of patients with Lemierre’s syndrome and COVID-19 complications [8, 9]. The compromised systemic condition caused by COVID-19 may contribute to the progression of Lemierre’s syndrome. Some reports have suggested an association between Epstein–Barr virus infection and Lemierre’s syndrome; however, the underlying mechanism remains unclear [1]. Therefore, COVID-19 may be associated with Lemierre’s syndrome.

Second, we observed the rapid development of a pulmonary artery pseudoaneurysm, which led to hemoptysis. Pulmonary pseudoaneurysms were observed on the 8^th^ but not the 6^th^ day of hospitalization (Figs. 1 and 2). Notably, these pseudoaneurysms emerged despite the patient receiving treatment for Lemierre’s syndrome and COVID-19. Hemoptysis rarely occurs in patients with Lemierre’s syndrome; additionally, pneumothorax is extremely rare. Further, patients with septic pulmonary embolisms rarely present with these symptoms [2, 10, 11]. However, pneumothorax commonly occurs in patients with COVID-19 [12, 13]. Thromboembolic complications, including pulmonary embolism, are commonly observed in patients with COVID-19; furthermore, COVID-19 is thought to induce systemic vascular lesions by mimicking vasculitis [14]. Although thrombotic complications are common, aneurysmal complications are very rare [15, 16]. Infections are the most common cause of pulmonary pseudoaneurysms [17]. In our patient’s case, COVID-19-induced pneumonia might have contributed to the development of pneumothorax; additionally, the comorbidity of Lemierre’s syndrome and COVID-19 led to the acute formation of a pulmonary pseudoaneurysm. The patient showed satisfactory recovery without any further episodes of hemoptysis after embolization. Our findings demonstrate the importance of early consideration of endovascular embolization in patients with hemoptysis associated with an infected pulmonary pseudoaneurysm.

Our findings demonstrate the importance of conducting comprehensive examinations for infectious diseases, including various culture tests, during the COVID-19 pandemic. Additionally, when managing patients with comorbid Lemierre’s syndrome and COVID-19, close monitoring is crucial given the potential risk of disease progression even with appropriate treatment.

## Data Availability

The dataset obtained and analyzed in this study is not publicly available because it contains personal information. This information is available from the corresponding author upon request.

## Abbreviations

CECT: Contrast-enhanced computed tomography
COVID-19: Coronavirus disease 2019
CT: Computed tomography
NBCA: N-butyl-2-cyanoacrylate
SARS-CoV-2: Severe acute respiratory syndrome coronavirus 2
